# Young Adults’ Sleep Duration on Work Days: Differences between East and West

**DOI:** 10.3389/fneur.2014.00081

**Published:** 2014-05-28

**Authors:** June C. Lo, Ruth L. F. Leong, Kep-Kee Loh, Derk-Jan Dijk, Michael W. L. Chee

**Affiliations:** ^1^Cognitive Neuroscience Laboratory, Neuroscience and Behavioral Disorders Program, Duke-NUS Graduate Medical School, Singapore, Singapore; ^2^Surrey Sleep Research Centre, Faculty of Health and Medical Sciences, University of Surrey, Guildford, UK

**Keywords:** work days, free days, sleep timing, sleep duration, social factors, Morningness–Eveningness preference, natural light

## Abstract

Human sleep schedules vary widely across countries. We investigated whether these variations were related to differences in social factors, Morningness–Eveningness (ME) preference, or the natural light–dark cycle by contrasting the sleep duration and timing of young adults (age: 18–35 years) on work and free days in Singapore (*n* = 1898) and the UK (*n* = 837). On work days, people in Singapore had later bedtimes, but wake times were similar to the UK sample, resulting in shorter sleep duration. In contrast, sleep duration on free days did not differ between the two countries. Shorter sleep on work days, without compensatory extra long sleep hours on free days, suggest greater demands from work and study in Singapore. While the two samples differed slightly in ME preference, the associations between eveningness preference and greater extension in sleep duration as well as delays in sleep timing on free days were similar in the two countries. Thus, differences in ME preference did not account for the differences in sleep schedules between the two countries. The greater variability in the photoperiod in the UK was not associated with more prominent seasonal changes in sleep patterns compared to Singapore. Furthermore, in the UK, daylight saving time did not alter sleep schedules relative to clock time. Collectively, these findings suggest that differences in social demands, primarily from work or study, could account for the observed differences in sleep schedules between countries, and that in industrialized societies, social zeitgebers, which typically involve exposure to artificial light, are major determinants of sleep schedules.

## Introduction

Epidemiological data have revealed considerable differences among countries in sleep duration and timing [e.g., Ref. (1)]. Compared to Western countries, people in Asia sleep less (2–6). A recent study showed that men and women in Japan, Korea, and Taiwan slept for less than 7 h on average, while all the 20 Western countries studied had an average of 7.08–8.04 h of sleep each night (3). In addition, people in Asia go to bed and wake up at later clock times than the Western nations (2). However, whether differences in sleep duration and timing are mostly contributed by social factors, Morningness–Eveningness (ME) preference, or natural light–dark cycle across countries remains to be formally studied.

The influence of social factors on sleep duration and timing is evident from comparing sleep schedules on work and free days. On work days, most people rise before their endogenous timing mechanism triggers spontaneous awakening. This is supported by the reliance on an alarm clock between 06:30 and 08:00 in 55% of respondents in a large-scaled survey (7). At night, availability of artificial light allows people to continue waking activities, such as watching TV (8), thereby delaying bedtime. Furthermore, artificial light in the evening has been shown to affect melatonin secretion and delay the biological clock and sleep (9, 10). As a result, sleep opportunity is reduced on work days. In contrast, on free days, with reduced demand from work and school and more social activities, individuals can sleep more in phase with their biological clock and until their sleep need is satisfied, resulting in a delay in sleep timing and an extension in sleep duration relative to work days (11, 12). Delays in bedtime, wake time, and mid-sleep time (the mid-point between sleep onset and offset), as well as increase in sleep duration on free days relative to work days have been observed in multiple countries (12–19). However, social demands vary across countries. Work hours in Asian countries, such as Japan, were longer than in Western countries on both weekdays and weekends (20). This perhaps can account for the shorter sleep duration in Asia.

Morningness–Eveningness preference is also associated with sleep duration and timing. Individuals can be categorized into morning, neither, or evening types based on their self-reports of preferred timing of various activities. Evening-type persons have later bedtime, wake time, and peak in mental and physical performance during the day (21). ME preference does not affect sleep duration averaged across the work week (21). However, greater eveningness preference is associated with shorter sleep on work days (22) but longer sleep on free days (22, 23). Thus, evening-type individuals accumulate greater sleep debt during work days that is compensated by lengthening their sleep on free days (22–25). ME preference may be associated with latitude (26), resulting in different sleep patterns across countries.

Countries at different latitudes also differ in the variability in the duration of scotopic period (the period between dusk/sunset and dawn/sunrise) and the timing of the natural light–dark cycle throughout the year. Light is a salient zeitgeber for entraining the non-24-h human circadian clock to the external environment (27). However, in Norway where scotopic period ranges between 0 and 24 h depending on time of year, wake time is only about 30 min later in winter than in summer (28), and seasonal changes in bedtime and sleep duration are minimal (28, 29). In regions near the equator where dusk and dawn times vary minimally over the year, sleep duration, and timing correspondingly stay relatively unchanged (28). Thus, latitude and the associated seasonal changes in natural light–dark cycle appear to have minimal influence on sleep (30).

Latitude can also influence sleep through the implementation of daylight saving time in some countries that are far away from the equator. To accommodate the seasonal changes in the natural light–dark cycle, these countries switch to daylight saving time in summer when clock time is shifted 1 h forward, whereas in winter, the clock is shifted back by 1 h to standard time. While the onset of daylight saving time does not affect sleep timing, at its offset, a delay in sleep timing has been reported (31). Thus, geographical differences in sleep patterns may also arise from these 1 h time changes in some countries.

Here, we first compared sleep duration and timing between two convenience samples of young adults, one from an Asian country located at the equator (1.3°N; Singapore) and the other from a Western country located at a high latitude (51.5°N; the UK). Secondly, we investigated whether these differences could be attributed to differences in social factors by comparing sleep on work and free days between the two countries. Thirdly, we examined whether ME preference could account for any differences in sleep between Singapore and the UK by testing whether the two countries differed in ME preference, and whether ME preference affected sleep duration and timing across the work week differentially in the two countries. Fourthly, we compared the duration of the scotopic period and the timing of the natural light–dark cycle between Singapore and the UK, and determined whether the natural light–dark cycle influenced sleep schedules. Fifthly, we investigated whether sleep patterns varied between daylight saving time and standard time in the UK (Singapore does not have daylight saving time). Finally, we compared the effect sizes of country, type of day, ME preference, and natural light–dark cycles on sleep duration and timing.

## Materials and Methods

### Participants

Convenience samples of young adults were acquired from Singapore and the UK. The Singapore sample consisted of 1898 volunteers (905 males) with an age range of 18–35 years (mean ± SD: 21.76 ± 2.41 years). The UK sample consisted of 837 volunteers (467 males) of the same age range (mean ± SD: 25.03 ± 4.07 years). The samples significantly differed in age (*t* = 21.62, *p* < 0.001) and gender distribution (χ*^2^* = 15.29, *p* < 0.001); hence, age and gender were included as covariates in those analyses testing the statistical significance of the country effect (refer to statistical analyses for further details). The majority (98.5%) of the participants in the Singapore sample were East Asians, while the UK sample consisted mainly of Europeans (Table S1 in Supplementary Materials).

### Procedures and materials

Participants in Singapore completed an online questionnaire on the Cognitive Neuroscience Laboratory website as part of the recruitment procedures for various sleep deprivation studies between December 2009 and December 2012. These studies were approved by the National University of Singapore Institutional Review Board. Participants were recruited through advertisements placed on the university intranet and posters on campus. Participants reported their sleep-wake timing and sleep duration on both weekdays and weekends. 99.5% of the participants were either studying or employed, allowing us to assume the timing and duration of sleep on weekdays and weekends to be equivalent to those typical of work and free days. To assess ME preference, participants completed the reduced Morningness-Eveningness Questionnaire [rMEQ; Ref. (32)], which consisted of five of the 19 items in the original version (21): (1) “Considering only your own ‘feeling best’ rhythm, at what time would you get up if you were entirely free to plan your day?” (2) “During the first half an hour after having woken in the morning, how tired do you feel?” (3) “At what time in the evening do you feel tired and as a result in need of sleep?” (4) “At what time of the day do you think that you reach your “feeling best” peak?” and (5) “One hears about ‘morning’ and ‘evening’ types of people. Which one of these types do you consider yourself to be?” To examine the reliability of the rMEQ, all of the participants in the Singapore sample with a valid email address were contacted in January 2013 for a more detailed assessment of their ME preference. Of these, 101 participants (41 males; mean age ± SD = 22.08 ± 2.41 years) completed the original MEQ. The correlation between rMEQ and MEQ scores was 0.84 (*p* < 0.001) – similar to that found in a previous study (33).

Participants in the UK were recruited for various sleep studies through email and posters on the University of Surrey campus, and advertisements on newspapers and radio. These studies received a favorable opinion from the University of Surrey Ethics Committee. During an extended screening session, participants reported their sleep timing and duration on both work and free days [see Ref. (19) for detailed procedures]. In addition to the participants screened between November 2008 and March 2010 as reported in Lazar et al. (19), the current sample included another 173 participants who were screened for other protocols between October 2004 and February 2008. All the participants completed all 19 items of the MEQ. We also calculated their rMEQ score, which highly correlated with their MEQ score (*r* = 0.90, *p* < 0.001) – again similar to a previous report (33).

For both samples, we used rMEQ score as a continuous measure of ME preference where higher scores indicate greater morningness preference.

Recruitment of participants in both countries occurred throughout the year (Table S2 in Supplementary Materials). For each participant, we calculated the duration of scotopic period (i.e., time elapsed between dusk and dawn), dusk time, dawn time, and mid-scotopic time (time of the mid-point of scotopic period) averaged across 30 days before they filled in the questionnaires. Data for both Singapore and the UK (London) were obtained from http://www.timeanddate.com.

### Statistical analyses

All statistical analyses were performed with SPSS 20.0 (IBM, Chicago, USA). To determine whether sleep duration, bedtime, wake time, and mid-sleep time differed between the two countries and varied across work and free days, we included country and type of day, as well as their interaction, into a general linear mixed model. Type of day was included as a repeated effect with a compound symmetry variance–covariance matrix being specified. To investigate whether sleep duration and timing were influenced by ME preference and whether this association differed between work and free days as well as countries, this factor and its two- and three-way interactions with type of day and country were also included into the statistical model. To test the contribution of the natural light–dark cycle on sleep, we added the duration of scotopic period, dusk time, dawn time, and mid-scotopic time into the model for sleep duration, bedtime, wake time, and mid-sleep time, respectively. Since the two samples differed in age and gender distribution, these two demographic variables were added as covariates. The subject effect was included as a random factor.

To compare the relative contribution of these factors to sleep schedules, for each term, we calculated Cohen’s *f^ 2^* as a measure of effect size (34, 35):

$$f^{2}=\left(u∕v\right)\times F$$

where *u* and *v* are respectively the numerator and denominator degrees of freedom of the *F* statistic used to determine the corresponding main or interaction effect in the general linear mixed model analysis. The cutoffs for small, medium, and large effect sizes are 0.02, 0.15, and 0.35, respectively. These cutoffs are different from those for Cohen’s *d* (0.20, 0.50, and 0.80 for small, medium, and large effects, respectively).

One of the items in the rMEQ was about preferred time to get up on free days, and thus, rMEQ (an independent variable) and self-report wake time (a dependent variable) may be measuring the same construct, resulting in a significant association. In view of this, we also conducted a second set of mixed model analyses without including this particular item in the computation of rMEQ score to determine the robustness of the contribution of ME preference to sleep.

For *post hoc* analyses, to examine significant country × type of day interactions, we contrasted the estimated marginal mean of the dependent variable on work and free days separately for Singapore and the UK. We also contrasted the estimated marginal mean of the dependent variable of the two countries separately on work and free days. These estimated marginal means were derived from the general linear mixed models, and thus, were already adjusted for age and gender. In addition, we used ANCOVA to test whether the change in sleep duration and timing from work to free days differed significantly between the two countries after controlling for the effects of age and gender.

To illustrate the significant type of day × ME preference interactions, we performed linear regression analyses where the dependent variable was regressed onto rMEQ score separately for work and free days. With the derived slope and intercept, we determined the values of the dependent variable when rMEQ score was minimum (i.e., 4 for the five-item rMEQ and 3 for the four-item version) and maximum (i.e., 25 and 20, respectively) to plot the regression lines. Furthermore, to determine the unique contribution of ME preference to the changes in sleep duration and timing from work to free days after controlling for the effects of age and gender, we used multiple regression analyses to derive the unstandardized regression coefficient (*B*) of rMEQ score.

We used a Chi-squared test to test whether the distribution of ME preference differed between Singapore and the UK. Note that in contrast to earlier findings (36), we did not observe any gender difference in ME preference (χ*^2^* = 2.37, *p* = 0.67). Hence, the differences in gender distribution between the two samples could not account for their differences in ME preference.

We used an independent-samples *t*-test to determine whether the two countries differed in their average ME preference. We also used independent-samples *t*-tests to examine whether the two countries differed in the natural light–dark cycle variables (duration of scotopic period, dusk time, dawn time, and mid-scotopic time) separately during daylight saving time and during standard time in the UK. The natural light–dark cycle data in 2012 were used for these analyses. Finally, we used independent-samples *t*-tests to determine whether sleep patterns were different at daylight saving time and at standard time in the UK.

## Results

### Sleep schedules differed across countries

Sleep duration averaged across work and free days was 0.51 h shorter in Singapore than in the UK (8.05 ± 0.02 h vs. 8.56 ± 0.04 h; main effect of country: *F* = 22.79, *p* < 0.001). This was due to a later bedtime in Singapore (00:39 ± 00:01 vs. 23:59 ± 00:02; *F* = 32.02, *p* < 0.001). The two countries did not differ in wake time (Singapore: 08:42 ± 00:01 vs. UK: 08:33 ± 00:02; *F* = 0.66, *p* = 0.42). As a result, average mid-sleep time was later in Singapore (04:41 ± 00:01 vs. 04:15 ± 00:02; *F* = 14.97, *p* < 0.001).

### Type of day moderated country differences in sleep schedules

**Figure 1:**
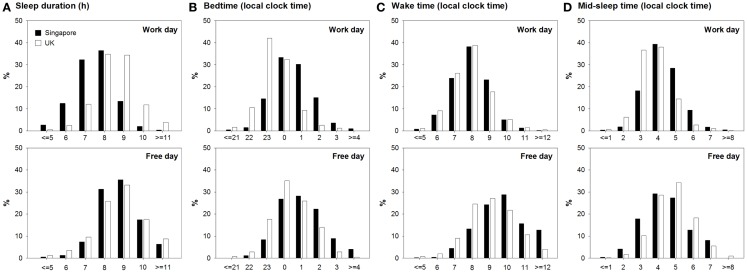
**Distributions of sleep duration and timing on work and free days in Singapore and the UK**. **(A)** Sleep duration, **(B)** bedtime, **(C)** wake time, and **(D)** mid-sleep time for Singapore and the UK are shown in black and white bars, respectively. Data on work and free days are respectively plotted in the upper and the lower panels.

Sleep duration was longer on free than on work days (8.71 ± 0.02 h vs. 7.91 ± 0.02 h; main effect of type of day: *F* = 326.18, *p* < 0.001). However, sleep extension from work to free days differed between the two countries. In Singapore, the majority of the participants reported sleeping 7–8 h on work days and 8–9 h on free days (Figure 1A). In contrast, in the UK, most individuals slept for 8–9 h on both work and free days (Figure 1A). This was confirmed by the significant country × type of day interaction on sleep duration (*F* = 28.55, *p* < 0.001), which revealed that individuals in Singapore slept less than their counterparts in the UK on work days. On free days, no such difference was found (Table 1). In other words, people in Singapore extended their sleep more on free days (main effect of country in ANCOVA: 1.29 ± 0.03 h vs. 0.32 ± 0.05 h; *F* = 259.39, *p* < 0.001).

**Table 1:** **Mean and standard error of sleep duration and timing on work and free days in Singapore and the UK**.

	Singapore			UK		
	Work day	Free day	*p*	Work day	Free day	*p*
Sleep duration (h)	7.41 (0.03)^a^	8.70 (0.03)	<0.001	8.40 (0.04)^a^	8.72 (0.04)	<0.001
Bedtime (local clock time)	00:23 (0:02)^a^	00:55 (0:02)^b^	<0.001	23:31 (0:02)^a^	00:28 (0:02)^b^	<0.001
Wake time (local clock time)	07:47 (0:02)	09:36 (0:02)^b^	<0.001	07:56 (0:03)	09:11 (0:03)^b^	<0.001
Mid-sleep time (local clock time)	04:05 (0:02)^a^	05:16 (0:02)^b^	<0.001	03:42 (0:03)^a^	04:49 (0:03)^b^	<0.001

*^a^Denotes contrast between Singapore and the UK on work days, where *p* < 0.001*.

*^b^Denotes contrast between Singapore and the UK on free days, where *p* < 0.001*.

*Listed values are estimated means and standard errors (in parentheses) from general linear mixed model*.

This was due to a smaller delay in bedtime (main effect of country in ANCOVA: 0.54 ± 0.02 h vs. 0.93 ± 0.04 h; *F* = 72.34, *p* < 0.001) and a greater delay in wake time on free days in Singapore (ANCOVA: 1.82 ± 0.03 vs. 1.25 ± 0.05 h; *F* = 82.15, *p* < 0.001) evidenced by the significant country × type of day interaction on bedtime and wake time (*F* = 5.36, *p* = 0.02 and *F* = 11.56, *p* < 0.001). These significant interactions also demonstrated that bedtime was significantly later in Singapore than in the UK on work days, but less so on free days (Table 1). In fact, on free days, the distributions of bedtime of the two countries both peaked at 00:00–01:00, while on work days, the peak shifted to 23:00–00:00 for the UK but not for Singapore (Figure 1B). Moreover, while people in the two countries woke up at similar times on work days, wake time on free days was significantly later in Singapore (Table 1). This can also be revealed by the similar distributions of wake time of the two countries on work days, which peaked at 08:00. In contrast, on free days, wake time peaked at 09:00–10:00 in Singapore but at 08:00–09:00 in the UK (Figure 1C).

Mid-sleep time was later on free days in both countries (main effect of type of day: *F* = 683.53, *p* < 0.001). The country × type of day interaction was not significant (*F* = 1.18, *p* = 0.28), indicating that the delay in mid-sleep time on free days relative to work days was similar in both countries (main effect of country in ANCOVA: 1.18 ± 0.02 h vs. 1.09 ± 0.04 h; *F* = 3.59, *p* = 0.06). In Singapore, mid-sleep time peaked at 04:00 on work days and shifted to 04:00–05:00 on free days, and in the UK, the peak of mid-sleep time was delayed from 03:00–04:00 on work days to 04:00–05:00 on free days (Figure 1D).

### The effect of morningness–eveningness preference varied with type of day but did not differ between countries

**Figure 2:**
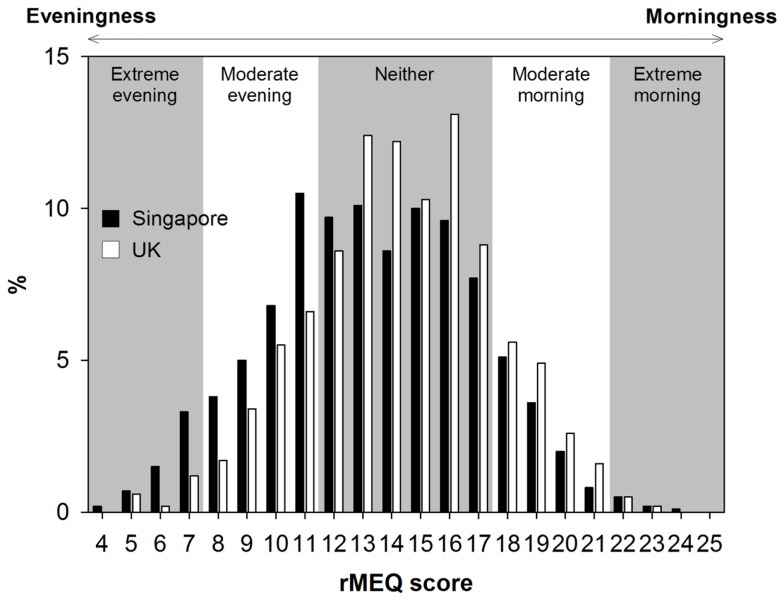
**Distributions of ME preference in Singapore and the UK**. The distributions of the total scores in the reduced Morningness–Eveningness Questionnaire (rMEQ) in Singapore and the UK are indicated, respectively, by the black and the white bars. Background is shaded based on the cutoff scores for the extreme, the moderate, and the neither types.

The distribution of ME preference was normal in Singapore, but shifted slightly to the right, toward more morning preference, in the UK (χ*^2^* = 67.81, *p* < 0.001; Figure 2). However, in both countries, the majority of the participants belonged to the neither type. Also, in both countries, there were more extreme evening-type than extreme morning-type individuals probably because of the age of our young adult samples. The two samples differed significantly in their rMEQ score but only by 0.93 points (maximum = 25; Singapore: 13.36 ± 3.56 vs. UK: 14.29 ± 3.21, *t* = 6.71, *p* < 0.001), and the size of the country effect was small (Cohen’s *d* = 0.26). We also found a small but significant difference in ME preference between Singapore and the UK when we used only four items of the rMEQ (maximum = 20; Singapore: 10.77 ± 3.06 vs. UK: 11.48 ± 2.74, *t* = 6.03, *p* < 0.001, Cohen’s *d* = 0.24; Figure S1 in Supplementary Materials).

As expected, ME preference was associated with bedtime (main effect: *F* = 772.19, *p* < 0.001), wake time (*F* = 866.45, *p* < 0.001), and mid-sleep time (*F* = 1093.91, *p* < 0.001). In contrast, ME preference was not significantly associated with sleep duration (*F* = 3.58, *p* = 0.059).

**Figure 3:**
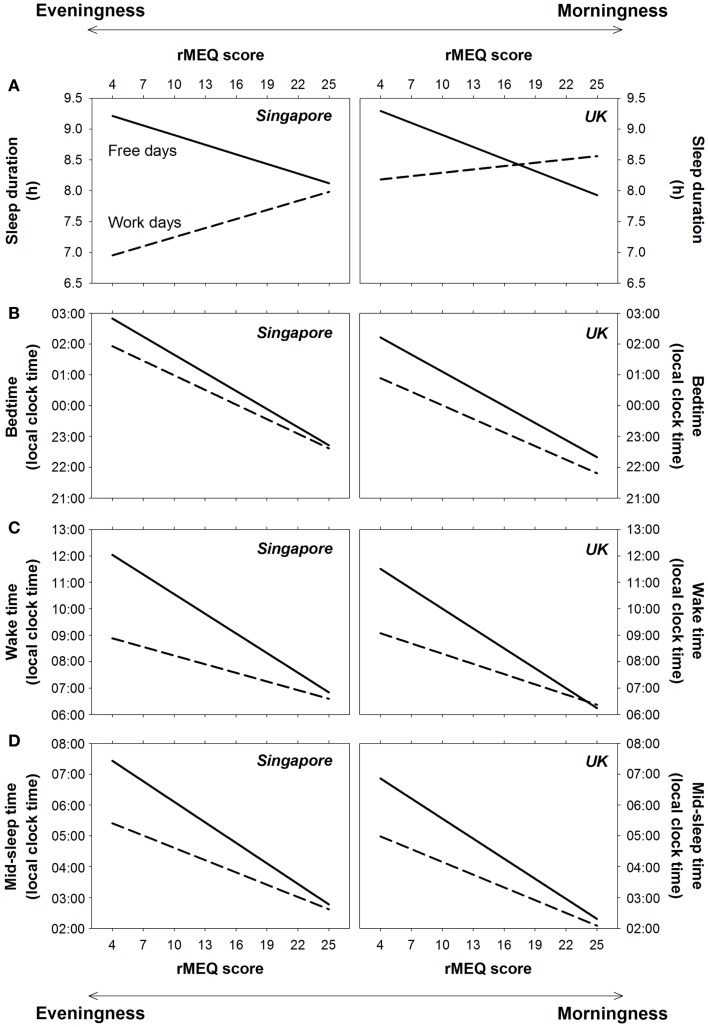
**Effects of ME preference on sleep on work days and free days in Singapore and the UK**. Regression lines for the effects of the reduced Morningness–Eveningness Questionnaire (rMEQ) score on **(A)** sleep duration, **(B)** bedtime, **(C)** wake time, and **(D)** mid-sleep time on work days (dashed lines) and free days (solid lines) for Singapore and the UK are plotted, respectively, in the left and the right panels.

However, when type of day was taken into account, we found significant associations of ME preference and sleep duration. Specifically, eveningness preference was associated with shorter sleep duration on work days but longer sleep duration and free days (type of day × ME preference: *F* = 136.41, *p* < 0.001). This implies that with increasing eveningness preference (decreasing rMEQ score), sleep was extended to a greater extent on free days relative to work days (*B* of rMEQ score = −0.10, *p* < 0.001 after controlling for the effects of age and gender). Although on work days, the difference in sleep duration between morning-type and evening-type individuals appeared to be smaller in the UK (Figure 3A), neither the country × type of day × ME preference interaction nor the country × ME preference interaction was statistically significant (*F* = 1.25 and 3.47, *p* = 0.26 and 0.06). Thus, ME preference moderated sleep duration on work and free days, as well as its change across a work week, similarly in the two countries.

Sleep extension on free days increased with eveningness preference because for morning-type individuals, bedtime, and wake time were similar on work and free days, whereas for evening-type individuals, the delay in wake time on free days was greater than that for bedtime (type of day × ME preference on bedtime: *F* = 37.13, *p* < 0.001; wake time: *F* = 259.85, *p* < 0.001; Figures 3B,C). The association between greater eveningness preference and greater delays in bedtime and wake time on free days was supported by regression analyses (*B* of rMEQ score = −0.04 and −0.14, *p* < 0.001 after controlling for age and gender). Similar patterns were found for Singapore and the UK (country × type of day × ME preference on bedtime and wake time: *F* = 0.00 and 1.21, *p* = 0.96 and 0.27; country × ME preference on bedtime and wake time: *F* = 3.47 and 0.00, *p* = 0.06 and 0.99; Figures 3B,C).

Because of the greater delay in bedtime and wake time among evening-type individuals, there was a greater delay in mid-sleep time on free days with increasing eveningness preference (type of day × ME preference: *F* = 192.65, *p* < 0.001). This association was also revealed by a regression analysis, which showed a significant contribution of rMEQ score to the change in mid-sleep time from work to free days after controlling for the effects of age and gender (*B* = −0.09, *p* < 0.001). The extent of delay in mid-sleep time on free days was similar for people in Singapore and the UK (country × type of day × ME preference: *F* = 0.60, *p* = 0.44; country × ME preference: *F* = 1.07, *p* = 0.30; Figure 3D).

Since one of the five questions in the rMEQ assessed preferred time to get up on free days, rMEQ would be expected to account for a considerable proportion of variance of self-report wake time and perhaps other sleep variables. To minimize the overlap of the rMEQ and the self-report sleep constructs, we re-ran the mixed model analyses but excluded this particular item from the computation of the rMEQ score. Results using the four-item and the five-item rMEQ were similar (Table 2 and Figure S2 in Supplementary Materials).

**Table 2:** **Contribution of country, type of day, Morningness–Eveningness preference, and the natural light–dark cycle to sleep duration and timing**.

	rMEQ (five items)			rMEQ (four items)		
	*F*	*p*	*f ^2^*	*F*	*p*	*f ^2^*
**SLEEP DURATION**						
Country	**22.79**	**<0.001**	**0.01**	**20.32**	**<0.001**	**0.01**
Type of day	**326.18**	**<0.001**	**0.12**	**276.19**	**<0.001**	**0.10**
ME preference	3.58	0.06	0.01	0.71	0.40	0.00
Duration of scotopic period	0.14	0.71	0.00	0.12	0.73	0.00
Country × type of day	**28.55**	**<0.001**	**0.01**	**28.19**	**<0.001**	**0.01**
Country × ME preference	3.47	0.06	0.00	2.10	0.15	0.00
Type of day × ME preference	**136.41**	**<0.001**	**0.05**	**96.59**	**<0.001**	**0.04**
Country × type of day × ME preference	1.25	0.26	0.00	0.59	0.44	0.00
Age	**5.56**	**0.02**	**0.00**	**7.95**	**0.01**	**0.00**
Gender	**4.74**	**0.03**	**0.00**	**4.32**	**0.04**	**0.00**
**BEDTIME**						
Country	**32.02**	**<0.001**	**0.01**	**34.08**	**<0.001**	**0.01**
Type of day	**195.11**	**<0.001**	**0.07**	**191.18**	**<0.001**	**0.07**
ME preference	**772.12**	**<0.001**	**0.28**	**690.59**	**<0.001**	**0.25**
Dusk time	1.06	0.30	0.00	1.31	0.25	0.00
Country × type of day	**5.36**	**0.02**	**0.00**	**6.02**	**0.01**	**0.00**
Country × ME preference	3.47	0.06	0.00	3.49	0.06	0.00
Type of day × ME preference	**37.13**	**0.00**	**0.01**	**29.83**	**0.00**	**0.01**
Country × type of day × ME preference	0.00	0.96	0.00	0.00	1.00	0.00
Age	**29.97**	**<0.001**	**0.01**	**40.75**	**<0.001**	**0.02**
Gender	**10.06**	**0.00**	**0.00**	**6.21**	**0.01**	**0.00**
**WAKE TIME**						
Country	0.66	0.42	0.00	1.57	0.21	0.00
Type of day	**806.87**	**<0.001**	**0.30**	**709.07**	**<0.001**	**0.26**
ME preference	**866.45**	**<0.001**	**0.32**	**597.72**	**<0.001**	**0.22**
Dawn time	0.05	0.82	0.00	0.15	0.70	0.00
Country × type of day	**11.56**	**0.00**	**0.00**	**10.59**	**0.00**	**0.00**
Country × ME preference	0.00	1.00	0.00	0.19	0.67	0.00
Type of day × ME preference	**259.85**	**<0.001**	**0.10**	**187.81**	**<0.001**	**0.07**
Country × type of day × ME preference	1.21	0.27	0.00	0.52	0.47	0.00
Age	**61.21**	**<0.001**	**0.02**	**78.69**	**<0.001**	**0.03**
Gender	1.11	0.29	0.00	0.25	0.62	0.00
**MID-SLEEP TIME**						
Country	**14.97**	**<0.001**	**0.01**	**17.31**	**<0.001**	**0.01**
Type of day	**683.53**	**<0.001**	**0.25**	**620.22**	**<0.001**	**0.23**
ME preference	**1093.91**	**<0.001**	**0.40**	**840.90**	**<0.001**	**0.31**
Mid-scotopic time	1.85	0.17	0.00	1.90	0.17	0.00
Country × type of day	1.18	0.28	0.00	0.88	0.35	0.00
Country × ME preference	1.07	0.30	0.00	1.59	0.21	0.00
Type of day × ME preference	**192.65**	**<0.001**	**0.07**	**143.05**	**<0.001**	**0.05**
Country × type of day × ME preference	0.60	0.44	0.00	0.25	0.62	0.00
Age	**58.02**	**<0.001**	**0.02**	**75.42**	**<0.001**	**0.03**
Gender	**5.70**	**0.02**	**0.00**	2.68	0.10	0.00

*Significant effects are highlighted in bold. *F* and *p* values were derived from general linear mixed model analyses (refer to Methods for details). Effect sizes were indicated by Cohen’s *f* ^2^. The original reduced Morningness–Eveningness Questionnaire (rMEQ) is comprised of 5 items, one of which probes the preferred time to get up on free days. This item and thus, the rMEQ score (the independent variable) would overlap considerably with self-report wake time (dependent variable). We conducted a second set of mixed model analyses, excluding this particular item in the computation of rMEQ score, to determine the robustness of the contribution of Morningness–Eveningness (ME) preference to sleep*.

### Natural light–dark cycle did not affect sleep duration or timing

**Figure 4:**
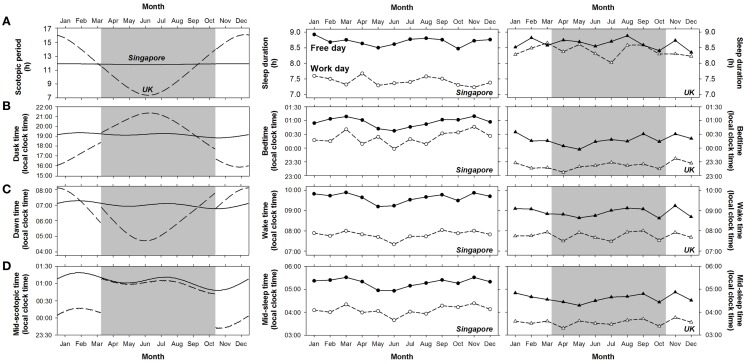
**Variation in the duration and timing of scotopic period and sleep in Singapore and the UK across a year**. Daily value of **(A)** the duration of scotopic period, **(B)** dusk time, **(C)** dawn time, and **(D)** mid-scotopic time in 2012 in Singapore (solid lines) and the UK (dashed lines) are shown in the left panel. Shaded areas indicate the daylight saving period in the UK. The monthly averages of **(A)** sleep duration, **(B)** bedtime, **(C)** wake time, and **(D)** mid-sleep time on work days (open symbols) and free days (filled symbols) for Singapore and the UK are plotted in the middle and the right panels, respectively. Considering the possible effects of daylight saving in the UK, we divided the daylight saving period in 2012 (25 March to 27 October) into seven roughly equal intervals and for each, derived the average sleep duration and timing. Similarly, we computed the monthly averages of the sleep variables for each of the five roughly equal intervals during standard time.

Since Singapore is closer to the equator than the UK, the natural light–dark cycle varies less across a year (left panel of Figure 4). Compared to Singapore, scotopic period is about 2.8 h longer in the UK during standard time because of the earlier dusk time and later dawn time (*t* = 6.21–36.42, *p* < 0.001; Table S3 in Supplementary Materials). In contrast, during daylight saving time, scotopic period is about 2.2 h shorter in the UK due to the later dusk time and earlier dawn time (*t* = 13.73–18.99, *p* < 0.001; Table S3 in Supplementary Materials). Compared to Singapore, mid-scotopic time in the UK is earlier by 1 h during standard time but by only 5 min during daylight saving time (*t* = 43.19 and 6.84, *p* < 0.001; Table S3 in Supplementary Materials).

Despite the differences between the two countries in the timing of the natural light–dark cycle, this had minimal effects on sleep. Firstly, variability in sleep duration and timing across a year in the two countries did not parallel the changes in the natural light–dark cycle (Figure 4). We did not observe greater variability in sleep schedules in the UK than in Singapore throughout the year (middle and right panels of Figure 4). In the UK, sleep duration, bedtime, wake time, and mid-sleep time were similar across daylight saving time in summer and standard time in winter, except for sleep timing on free days, which was about 10 min *earlier* during daylight saving time (*t* = 2.00–2.40, *p* < 0.05; Table 3). These effects of daylight saving were small (all Cohen’s *d* < 0.17, Table 3) and in fact, opposite to the direction predicted if the timing of the natural light–dark cycle were to affect sleep times. Specifically, when local clock time is shifted forward by 1 h during daylight saving time, if sleep timing is tightly coupled with the timing of the natural light–dark cycle, bedtime, wake time, and mid-sleep time should all be *delayed* by 1 h (in local clock time). Instead, the slightly *earlier* sleep timings during daylight saving time suggest that sleep schedules of individuals in the UK are more coupled with timing of the clock rather than that of the sun. In addition, there was no abrupt change in either the duration or the timing of sleep (expressed as local clock time) at the onset and offset of daylight saving time in the UK (right panel of Figure 4).

**Table 3:** **Effects of daylight saving on sleep duration and timing in the UK**.

	Daylight saving time	Standard time	*t*	*p*	*d*
**SLEEP DURATION (H)**					
Work day	3.38 ± 0.05	8.38 ± 0.06	0.12	0.90	0.01
Free day	8.62 ± 0.06	8.66 ± 0.07	0.52	0.61	0.04
**BEDTIME (LOCAL CLOCK TIME)**					
Work day	23:18 ± 0:03	23:25 ± 0:03	1.61	0.11	0.11
Free day	00:13 ± 0:03	00:23 ± 0:03	**2.00**	**0.05**	**0.14**
**WAKE TIME (LOCAL CLOCK TIME)**					
Work day	07:41 ± 0:03	07:48 ± 0:03	1.58	0.12	0.11
Free day	08:49 ± 0:03	09:03 ± 0:04	**2.22**	**0.03**	**0.15**
**MID-SLEEP TIME (LOCAL CLOCK TIME)**					
Work day	03:30 ± 0:02	03:37 ± 0:03	1.85	0.07	0.13
Free day	04:32 ± 0:03	04:43 ± 0:03	**2.40**	**0.02**	**0.17**

**t* = *t*-value of independent-samples *t*-test*.

**d* = Cohen’s *d* of the effects of daylight saving*.

*Significant effects are highlighted in bold*.

Secondly, there was no significant effect of the duration of scotopic period, dusk time, dawn time, or mid-scotopic time on respectively sleep duration (*F* = 0.14, *p* = 0.71), bedtime (*F* = 1.06, *p* = 0.30), wake time (*F* = 0.05, *p* = 0.82), or mid-sleep time (*F* = 1.85, *p* = 0.17). Pearson correlations between the natural light–dark cycle and the sleep variables on work and free days in the two countries were not significant, except for the associations on work days in Singapore between dusk time and bedtime (*r* = −0.13, *p* < 0.001), and between mid-scotopic period and mid-sleep time (*r* = −0.08, *p* < 0.001; Table S4 in Supplementary Materials). However, it is important to note that even for these significant associations, the natural light–dark cycle only accounted for a mere 1.6 and 0.7% of the variance of sleep duration and timing, and that these statistically significant associations were likely due to the large sample size.

### Social influences were stronger than the effects of the natural light–dark cycle on sleep

Social influences, as revealed by the type of day effect, on sleep were noticeable throughout the year not only in Singapore, but also in the UK where variability in the natural light–dark cycle is more prominent (Figure 4). The effect size of natural light–dark cycle on all the sleep variables was negligible (all Cohen’s *f^ 2^* = 0.00; Table 2). In contrast, type of day had moderate effects on wake time and mid-sleep time (Cohen’s *f^ 2^* = 0.23 to 0.30), and relatively smaller effects on bedtime and sleep duration (Cohen’s *f^ 2^* = 0.07 to 0.12).

Morningness–Eveningness preference had stronger associations with the timing of sleep, i.e., bedtime, wake time, and mid-sleep time (Cohen’s *f ^2^* = 0.22 to 0.40) than with sleep duration (Cohen’s *f ^2^* = 0.00). However, its relationship with sleep duration became apparent when type of day was taken into account (Cohen’s *f ^2^* of type of day × ME preference = 0.04–0.05). Results were similar regardless of whether the item about preferred time to get up on free days was removed from the computation of the rMEQ score (Table 2).

Despite the considerable variation in sleep duration and timing between the two countries, the size of the country effect was rather small (Cohen’s *f ^2^* = 0.00 to 0.01).

## Discussion

We surveyed sleep in over 2,700 young adults in Singapore and the UK and found that sleep duration and timing differed between these two countries. In both, participants slept later and for longer on free days than on work days. However, changes in sleep timing and duration across the work week were dissimilar, reflecting different social influences on sleep. In both countries, ME preference similarly modulated the change in sleep schedules from work to free days. In contrast, we found limited effects of the natural light–dark cycle on sleep schedules. In particular, despite the much greater fluctuation in the duration of scotopic period and the timing of the natural light–dark cycle in the UK, we did not observe corresponding greater changes in sleep schedules across the year than in equatorial Singapore. Furthermore, daylight saving did not alter sleep schedules in the UK. These results remained unchanged after controlling for between country differences in ME preference, age, and gender.

### Differences in sleep schedules across countries may be attributed to differing social factors

Our observation concerning shorter sleep duration in Singapore than in the UK is in accordance with the shorter sleep duration in Asian than in Western countries as previously reported (2–6).

Here, with more detailed analyses of sleep duration and timing separately on work and free days, we showed that sleep duration was longer on free than on work days in both Singapore and the UK, consistent with findings from other countries (12–19). Nevertheless, sleep was extended more on free days relative to work days in Singapore than in the UK. This is a result of the shorter sleep duration in Singapore on work days. In contrast, sleep duration on free days did not differ between the two countries. These findings could on one hand, suggest that Singaporeans may have lower sleep need and can better cope with sleep restriction during work days without sleeping longer on free days. Alternatively, if sleep need does not vary across countries, our findings could also indicate that Singaporeans are chronically sleep-deprived. The second interpretation is favored, since preliminary evidence suggests that sleep need does not vary much across countries. For example, in a recent study, self-reported sleep need ranged from 7.7 to 8.4 h in 15 countries in the Asia-Pacific, while sleep duration showed more variability (from 6.3 to 7.5 h) (37).

We cannot rule out the possibility that young adults in Singapore napped more often than their counterparts in the UK in order to compensate for their shorter sleep duration at night. However, there is no tradition of napping among adults in Singapore and initial epidemiological evidence showed that countries where residents reported short nocturnal sleep duration did not have a greater proportion of regular nappers (1). Also, our results were not likely a consequence of the differences in temperature between the two countries because of the frequent use of air-conditioning throughout the year in Singapore and heaters in winter in the UK.

Sleep duration on free days was 8.7 h in young people in both countries. This was shorter than the maximal sleep capacity of this age group. Specifically, a recent study (38) reported that young adults with an average habitual sleep duration of 8.5 h could still sleep for 12.2 and 10.6 h in the first two 24-h periods of 16-h sleep opportunity. Thus, it was physiologically possible for our two samples to sleep more than 8.7 h on free days. We speculate that sleep duration on free days was far from reaching the maximal sleep capacity because of work- and study-related activities even on free days. For example, in an international time use survey, people reported spending 1–2 h each day during weekends on job- and study-related activities in Western countries, but about 3 h in Japan and Korea (20). This difference could, in fact, explain why sleep duration was not longer on free days in Singapore than in the UK. Similarly, shorter sleep duration in Singapore on work days could be due to longer work hours in Asia than in the West (20).

Sleep duration in both Singapore and the UK reported here appears to be slightly longer than the estimates for the East and the West from other surveys [e.g., Ref. (6)]. This is likely due to the use of a wider age range, as well as the inclusion of older adults and retirees in previous studies.

Other than sleep duration, the timing of sleep also differed between the two countries. Even after correcting for the younger age and greater eveningness preference of Singaporean participants, we continued to observe later bedtime and mid-sleep times on both work and free days in this group. Later sleep timings in Asia than in Western countries have been reported previously (2). Such a sleep schedule becomes problematic on work days when people in Singapore go to bed later but wake up at the same time as their counterparts in the UK, resulting in shorter sleep duration. The later bedtimes on work days in Singapore could also be due to greater demands from work and study. For example, classes end typically by 18:00 in the UK, while in Singapore, some lectures actually start at 18:00 and tutorials can end as late as 23:30, delaying bedtime.

Our data replicate earlier findings for later bedtime, wake time, and mid-sleep time on free days relative to work days (12–19). To these findings, we add the observation that the delay in sleep timing from work to free days differs between the two countries. On free days, bedtime was delayed less and wake time delayed more in Singapore than in the UK, thereby increasing the sleep opportunity more for Singaporeans.

The prominent differences in sleep duration and timing on work and free days, as well as their changes across the work week, observed between Singapore and the UK clearly reveal that differences in social influences can account for the differences in sleep schedules between the two countries.

Chronic sleep restriction has negative consequences on health (3, 39) and cognitive performance (35, 40). Whether impaired health and cognitive functions are more prevalent in countries where the populace is chronically sleep restricted remains to be investigated.

### Morningness–eveningness preference modulated changes in sleep schedule from work to free days similarly in both countries

We replicated earlier findings that evening preference is associated with later sleep timing (18, 22–25, 41). Evening preference is also characterized by greater delays in bedtime, wake time, and mid-sleep time, as well as more extended sleep on free days (22–25). The association between ME preference and the length of sleep extension on free days may be attributed to individual differences in the timing and the period of the endogenous circadian rhythm. Specifically, eveningness preference has been linked to later circadian rhythms [e.g., Ref. (21, 41–43)], which, in turn, are associated with longer circadian period and greater sleep extension on free days (12).

Importantly, we showed for the first time that ME preference modulates changes in sleep schedules from work to free days similarly in Singapore and the UK, suggesting that these findings might be generalizable to other industrialized countries. Furthermore, across countries, evening-type individuals appear to restrict their sleep on work days and delay their mid-sleep time on free days to similar extents. Hence, evening-type individuals appear prone to sleep curtailment and circadian misalignment. This could adversely affect their cognitive performance and health in the long term (35, 40, 44).

### Sleep schedules were relatively unaffected by the natural light–dark cycle

Although light is a prominent zeitgeber (27), the natural light–dark cycle did not significantly affect sleep schedules, perhaps because of the pervasiveness of strong artificial light in the test countries. The effects of the natural light–dark cycle on sleep duration and timing were negligible compared to the contribution of social factors and ME preference. Contrary to earlier findings (28), greater seasonal variation in the duration and timing of the natural light–dark cycle in a country at high latitude did not result in more variable sleep patterns throughout the year. In fact, we found limited seasonal change in sleep in the UK – a finding that has also been observed in Norway (29). Furthermore, in the UK, changes between daylight saving and standard times were not associated with abrupt changes in sleep duration and timing, although others have reported disruptive effects of daylight saving on sleep (31).

Discrepancies between our findings and others could be due to the nature of our samples, which consisted mainly of students who spend most of their time indoor and have more exposure to electrical rather than natural light. While some have suggested that it is the amount of time spent outdoor in broad daylight that has significant impact on sleep timing (11), the effects of electrical artificial light are not negligible (9, 10). In industrialized societies where electrical lighting is abundant, exposure to sunlight during daytime is reduced, light exposure after sunset is increased, circadian timing is delayed, and endogenous circadian clock is less synchronized with the natural light–dark cycle relative to an environment with only natural light exposure (10). In addition, compared with individuals residing in rural areas, urban dwellers have less exposure to natural light and later sleep schedules (45). Apart from its effects on sleep timing, electrical lighting may also be a cause of insufficient sleep in industrialized societies (46).

The absence of a significant effect of the natural light–dark cycle on sleep timing and duration suggests that the significant country effects on sleep in our two young adults samples cannot be attributed to differences in the natural light–dark cycle between Singapore and the UK. In addition, together with the prominent influence of social factors on sleep schedules, these findings are consistent with the epidemiological evidence that natural light has a smaller influence on the circadian clock in individuals residing in larger cities where social influences and the behavioral light–dark cycle may have stronger impact (47).

### Limitations

The data presented here were obtained with convenience sampling during the screening phase of different sleep protocols in Singapore and the UK. Recruitment and screening methodologies were not identical for the two samples. Firstly, sleep studies were advertised primarily on campus in Singapore as opposed to the additional use of newspaper and radio in the UK. This could account for the younger mean age of the Singapore sample, which included mainly undergraduates and postgraduates. We addressed these differences in demographics by including age as a covariate in all the analyses testing the country contrast. Furthermore, restricting our analyses to a narrower age range (i.e., 18–25) and individuals who were in full-time education (*n* = 1737 in Singapore; *n* = 100 in the UK) yielded similar findings for all the main and interaction effects of country, type of day, ME preference, and natural light–dark cycle variables (Table S5 in Supplementary Materials). These two more homogenous samples allowed us to address a second methodological difference – the assessment of sleep schedules on weekdays and weekends in Singapore vs. on work and free days in the UK. The results from the full and the homogenized samples were similar, indicating that for most young adults, sleep on weekdays is similar to sleep on work days, and so is sleep on weekends and free days. Thirdly, participants filled in the questionnaires online outside the sleep laboratory in Singapore but in the presence of an experimenter in the UK. However, in both countries, participants were not aware of the selection criteria regarding their sleep schedules and ME preference for the advertised sleep studies. Therefore, greater demand characteristics due to the presence of an experimenter in the UK were unlikely.

Like many large-scale epidemiological studies, sleep timing, and duration were assessed by questionnaires here. However, subjective reports over-estimate actual sleep duration [e.g., Ref. (48)]. Future studies would do well to determine whether our findings are replicated with actigraphically and polysomnographically assessed sleep.

While people in Singapore and the UK slept longer on free days, our data did not allow us to assess the effectiveness of this practice in reducing and clearing the sleep debt accumulated over work days. However, after multiple nights of partial sleep deprivation, when participants were given longer sleep opportunities, cognitive performance improved, and daytime sleep latency increased, indicating a reduction in sleep pressure (49). The amount of sleep required on free days to completely clear sleep debt and its association with the extent of sleep loss on work days remain to be determined.

In conclusion, sleep duration and timing varied between two urbanized Asian and Western countries. Our data suggest that differences in sleep schedules across urbanized societies are better explained by the variability in social demands rather than differences in daylight duration and timing. Moreover, in industrialized societies, sleep duration and timing are more influenced by social zeitgebers, which include exposure to artificial light, than natural light.

## Conflict of Interest Statement

The authors declare that the research was conducted in the absence of any commercial or financial relationships that could be construed as a potential conflict of interest.

## Supplementary Materials

The Supplementary Material for this article can be found online at http://www.frontiersin.org/Journal/10.3389/fneur.2014.00081/abstract

## References

[1] Soldatos CR Allaert FA Ohta T Dikeos DG How do individuals sleep around the world? Results from a single-day survey in ten countries. Sleep Med (2005) 6:5–13 10.1016/j.sleep.2004.10.006 15680289

[2] AC Nielsen. Consumers in Asia Pacific: Our Sleeping Patterns. (2005). Available from: http://jp.en.nielsen.com/news/documents/AsiaPacificSleepingPatternsReport.pdf

[3] Steptoe A Peacey V Wardle J Sleep duration and health in young adults. Arch Intern Med (2006) 166:1689–92 10.1001/archinte.166.16.1689 16983045

[4] Fisher K Robinson J Average weekly time spent in 30 basic activities across 17 countries. Soc Indic Res (2009) 93:249–54 10.1007/s11205-008-9372-y

[5] Gradisar M Gardner G Dohnt H Recent worldwide sleep patterns and problems during adolescence: a review and meta-analysis of age, region, and sleep. Sleep Med (2011) 12:110–8 10.1016/j.sleep.2010.11.008 21257344

[6] National Sleep Foundation. Bedroom Poll: Summary of Findings. (2013). Available from: http://sleepfoundation.org/sites/default/files/RPT495a.pdf

[7] Roenneberg T Kuehnle T Juda M Kantermann T Allebrandt K Gordijn M Epidemiology of the human circadian clock. Sleep Med Rev (2007) 11:429–38 10.1016/j.smrv.2007.07.005 17936039

[8] Basner M Dinges DF Dubious bargain: trading sleep for Leno and Letterman. Sleep (2009) 32:747–52 19544750 10.1093/sleep/32.6.747 PMC2690561

[9] Santhi N Thorne HC Van Der Veen DR Johnsen S Mills SL Hommes V The spectral composition of evening light and individual differences in the suppression of melatonin and delay of sleep in humans. J Pineal Res (2012) 53:47–59 10.1111/j.1600-079X.2011.00970.x 22017511

[10] Wright KP Jr Mchill AW Birks BR Griffin BR Rusterholz T Chinoy ED Entrainment of the human circadian clock to the natural light-dark cycle. Curr Biol (2013) 23:1554–8 10.1016/j.cub.2013.06.039 23910656 PMC4020279

[11] Roenneberg T Wirz-Justice A Merrow M Life between clocks: daily temporal patterns of human chronotypes. J Biol Rhythms (2003) 18:80–90 10.1177/0748730402239679 12568247

[12] Lazar AS Santhi N Hasan S Lo JC Johnston JD Von Schantz M Circadian period and the timing of melatonin onset in men and women: predictors of sleep during the weekend and in the laboratory. J Sleep Res (2013) 22:155–9 10.1111/jsr.12001 23216995

[13] Johns MW Gay TJ Goodyear MD Masterton JP Sleep habits of healthy young adults: use of a sleep questionnaire. Br J Prev Soc Med (1971) 25:236–41 5160432 10.1136/jech.25.4.236 PMC478664

[14] Monk TH Buysse DJ Rose LR Hall JA Kupfer DJ The sleep of healthy people: a diary study. Chronobiol Int (2000) 17:49–60 10.1081/CBI-100101031 10672433

[15] Zavada A Gordijn MC Beersma DG Daan S Roenneberg T Comparison of the Munich chronotype questionnaire with the Horne-Ostberg’s morningness-eveningness score. Chronobiol Int (2005) 22:267–78 10.1081/CBI-200053536 16021843

[16] Basner M Fomberstein KM Razavi FM Banks S William JH Rosa RR American time use survey: sleep time and its relationship to waking activities. Sleep (2007) 30:1085–95 17910380 10.1093/sleep/30.9.1085 PMC1978395

[17] Tsui YY Wing YK A study on the sleep patterns and problems of university business students in Hong Kong. J Am Coll Health (2009) 58:167–76 10.1080/07448480903221418 19892654

[18] Soehner AM Kennedy KS Monk TH Circadian preference and sleep-wake regularity: associations with self-report sleep parameters in daytime-working adults. Chronobiol Int (2011) 28:802–9 10.3109/07420528.2011.613137 22080786 PMC4143130

[19] Lazar AS Slak A Lo JC Santhi N Von Schantz M Archer SN Sleep, diurnal preference, health, and psychological well-being: a prospective single-allelic-variation study. Chronobiol Int (2012) 29:131–46 10.3109/07420528.2011.641193 22324552

[20] Fisher K Robinson J Daily Routines in 22 Countries: Diary Evidence of Average Daily Time Spent in Thirty Activities. (2010). Available from: http://www-2009.timeuse.org/research/docs/technical-papers/2010-01.pdf

[21] Horne JA Ostberg O A self-assessment questionnaire to determine morningness-eveningness in human circadian rhythms. Int J Chronobiol (1976) 4:97–110 1027738

[22] Giannotti F Cortesi F Sebastiani T Ottaviano S Circadian preference, sleep and daytime behaviour in adolescence. J Sleep Res (2002) 11:191–9 10.1046/j.1365-2869.2002.00302.x 12220314

[23] Roepke SE Duffy JF Differential impact of chronotype on weekday and weekend sleep timing and duration. Nat Sci Sleep (2010) 2010:213–20 20890372 10.2147/NSS.S12572 PMC2947028

[24] Taillard J Philip P Bioulac B Morningness/eveningness and the need for sleep. J Sleep Res (1999) 8:291–5 10.1046/j.1365-2869.1999.00176.x 10646169

[25] Korczak AL Martynhak BJ Pedrazzoli M Brito AF Louzada FM Influence of chronotype and social zeitgebers on sleep/wake patterns. Braz J Med Biol Res (2008) 41:914–9 10.1590/S0100-879X2008005000047 18982197

[26] Randler C Morningness-eveningness comparison in adolescents from different countries around the world. Chronobiol Int (2008) 25:1017–28 10.1080/07420520802551519 19005902

[27] Duffy JF Czeisler CA Effect of light on human circadian physiology. Sleep Med Clin (2009) 4:165–77 10.1016/j.jsmc.2009.01.004 20161220 PMC2717723

[28] Friborg O Bjorvatn B Amponsah B Pallesen S Associations between seasonal variations in day length (photoperiod), sleep timing, sleep quality and mood: a comparison between Ghana (5 degrees) and Norway (69 degrees). J Sleep Res (2012) 21:176–84 10.1111/j.1365-2869.2011.00982.x 22074234

[29] Sivertsen B Overland S Krokstad S Mykletun A Seasonal variations in sleep problems at latitude 63 degrees −65 degrees in Norway: the Nord-Trondelag Health Study, 1995-1997. Am J Epidemiol (2011) 174:147–53 10.1093/aje/kwr052 21555717

[30] Borisenkov MF Perminova EV Kosova AL Chronotype, sleep length, and school achievement of 11- to 23-year-old students in northern European Russia. Chronobiol Int (2010) 27:1259–70 10.3109/07420528.2010.487624 20653453

[31] Kantermann T Juda M Merrow M Roenneberg T The human circadian clock’s seasonal adjustment is disrupted by daylight saving time. Curr Biol (2007) 17:1996–2000 10.1016/j.cub.2007.10.025 17964164

[32] Adan A Almirall H Horne and Ostberg morningness eveningness questionnaire: a reduced scale. Pers Indiv Differ (1991) 12:241–53 10.1016/0191-8869(91)90110-W

[33] Di Milia L Adan A Natale V Randler C Reviewing the psychometric properties of contemporary circadian typology measures. Chronobiol Int (2013) 30:1261–71 10.3109/07420528.2013.817415 24001393

[34] Cohen J Statistical Power Analysis for the Behavioral Sciences. Hillsdale, NJ: Lawrence Erlbaum Associates (1988).

[35] Lo JC Groeger JA Santhi N Arbon EL Lazar AS Hasan S Effects of partial and acute total sleep deprivation on performance across cognitive domains, individuals and circadian phase. PLoS One (2012) 7:e45987 10.1371/journal.pone.0045987 23029352 PMC3454374

[36] Adan A Natale V Gender differences in morningness-eveningness preference. Chronobiol Int (2002) 19:709–20 10.1081/CBI-120005390 12182498

[37] AIA. AIA Healthy Living Index Survey: Regional Findings Across 15 Markets. (2011). Available: http://www.aia.com/en/about-aia/about-us/community/healthy-living/news.html

[38] Klerman EB Dijk DJ Age-related reduction in the maximal capacity for sleep: implications for insomnia. Curr Biol (2008) 18:1118–23 10.1016/j.cub.2008.06.047 18656358 PMC2582347

[39] Luyster FS Strollo PJ Jr Zee PC Walsh JK Sleep: a health imperative. Sleep (2012) 35:727–34 22654183 10.5665/sleep.1846 PMC3353049

[40] Kronholm E Sallinen M Era P Suutama T Sulkava R Partonen T Psychomotor slowness is associated with self-reported sleep duration among the general population. J Sleep Res (2011) 20:288–97 10.1111/j.1365-2869.2010.00899.x 21129054

[41] Bailey SL Heitkemper MM Morningness-eveningness and early-morning salivary cortisol levels. Biol Psychol (1991) 32:181–92 10.1016/0301-0511(91)90009-6 1790270

[42] Kerkhof GA Van Dongen HP Morning-type and evening-type individuals differ in the phase position of their endogenous circadian oscillator. Neurosci Lett (1996) 218:153–6 10.1016/S0304-3940(96)13140-2 8945751

[43] Duffy JF Dijk DJ Hall EF Czeisler CA Relationship of endogenous circadian melatonin and temperature rhythms to self-reported preference for morning or evening activity in young and older people. J Investig Med (1999) 47:141–50 10198570 PMC8530273

[44] Roenneberg T Allebrandt KV Merrow M Vetter C Social jetlag and obesity. Curr Biol (2012) 22:939–43 10.1016/j.cub.2012.03.038 22578422

[45] Carvalho FG Hidalgo MP Levandovski R Differences in circadian patterns between rural and urban populations: an epidemiological study in countryside. Chronobiol Int (2014) 31:442–9 10.3109/07420528.2013.846350 24397277

[46] Czeisler CA Perspective: casting light on sleep deficiency. Nature (2013) 497:S13 10.1038/497S13a 23698501

[47] Roenneberg T Kumar CJ Merrow M The human circadian clock entrains to sun time. Curr Biol (2007) 17:R44–5 10.1016/j.cub.2006.12.011 17240323

[48] Lauderdale DS Knutson KL Yan LL Liu K Rathouz PJ Self-reported and measured sleep duration: how similar are they? Epidemiology (2008) 19:838–45 10.1097/EDE.0b013e318187a7b0 18854708 PMC2785092

[49] Rupp TL Wesensten NJ Bliese PD Balkin TJ Banking sleep: realization of benefits during subsequent sleep restriction and recovery. Sleep (2009) 32:311–21 19294951 10.1093/sleep/32.3.311 PMC2647785

