# Supplementary material for: Young Adults’ Sleep Duration on Work Days: Differences between East and West
Source: Front Neurol. 2014 May 28;5:81. doi: 10.3389/fneur.2014.00081 (PMC4036075; doi:10.3389/fneur.2014.00081)

FIGURE S1 Distributions of ME preference in Singapore and the UK. Here, the item about preferred time to get up on free days was removed from the computation of the reduced Morningness-Eveningness Questionnaire (rMEQ) score. The distributions of rMEQ scores in Singapore and the UK are indicated respectively by the black and the white bars.

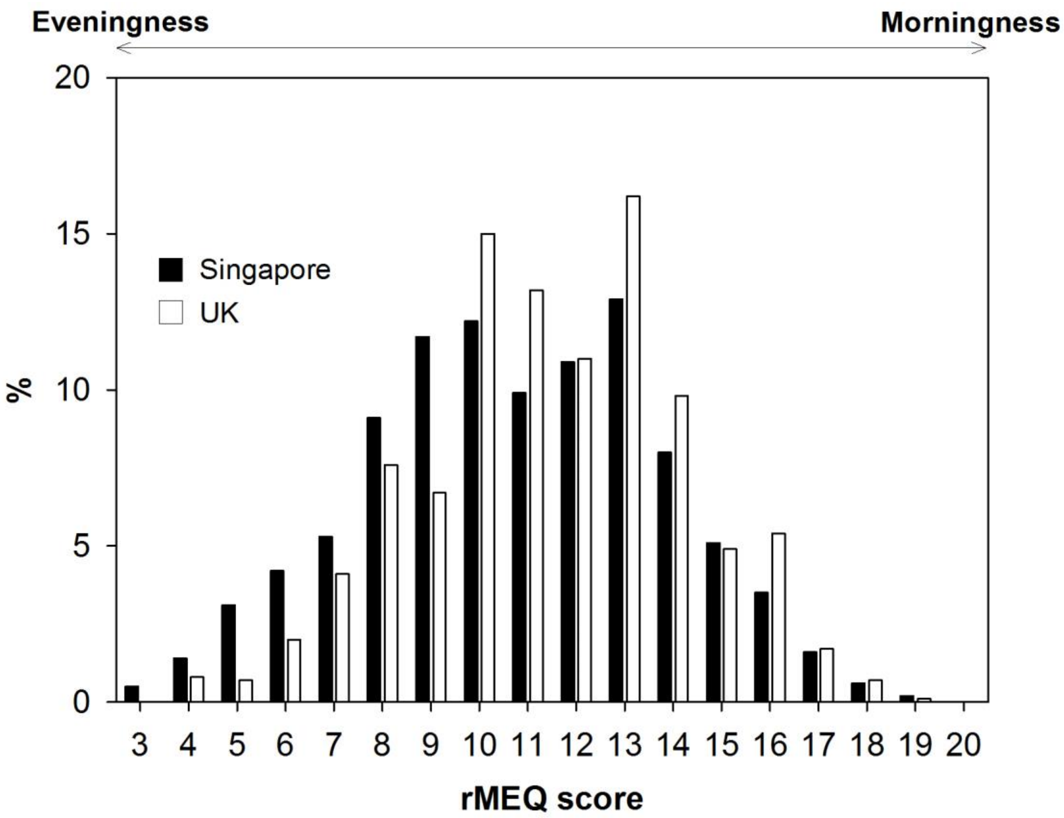

FIGURE S2 Effects of ME preference on sleep on work days and free days in Singapore and the UK. Here, the item about preferred time to get up on free days was removed from the computation of the reduced Morningness-Eveningness Questionnaire (rMEQ) score. Regression lines for the effects of rMEQ score on (A) sleep duration, (B) bedtime, (C) wake time, and (D) mid-sleep time on work days (dashed lines) and free days (solid lines) for Singapore and the UK are plotted respectively in the left and the right panels.

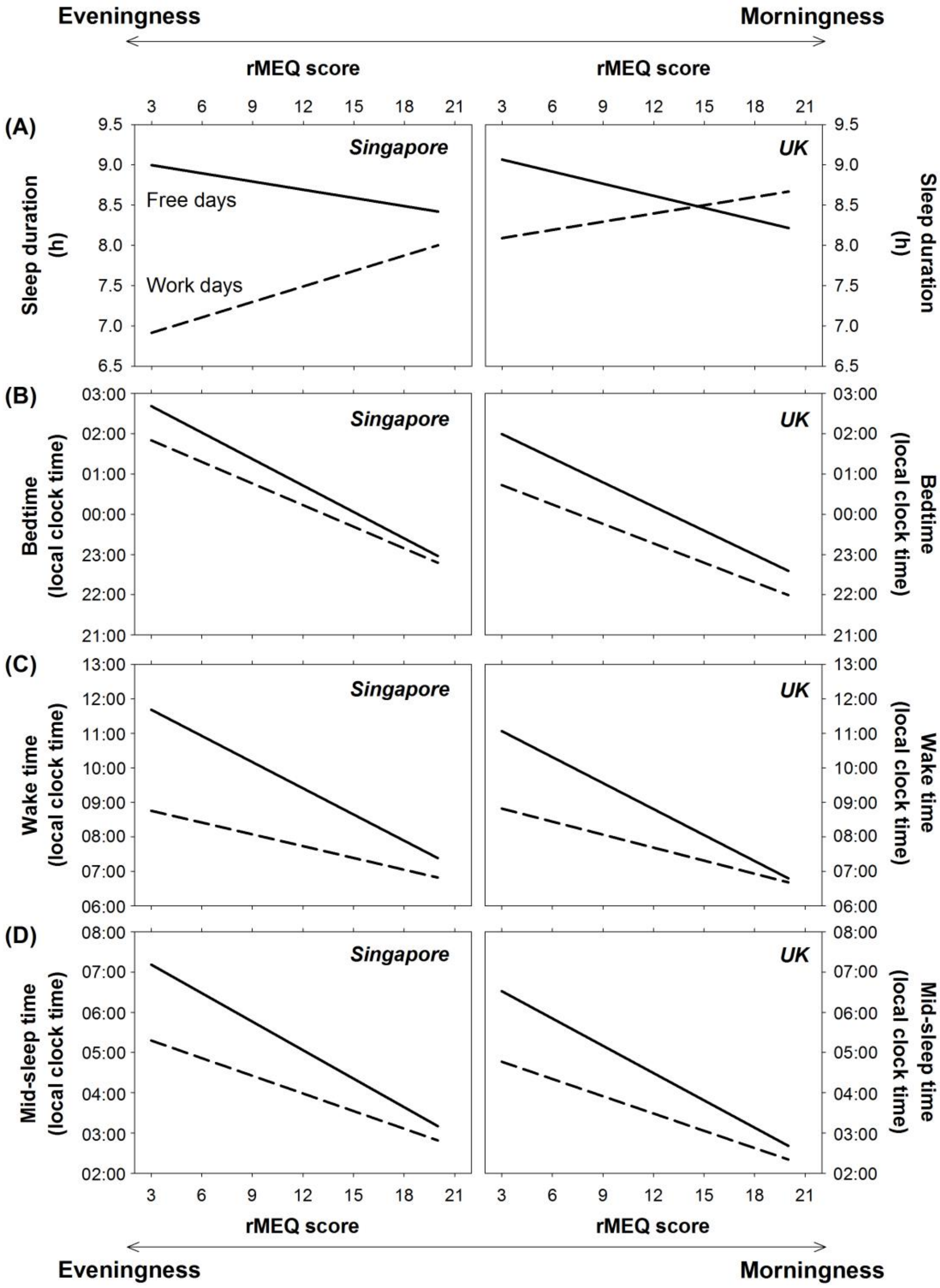

Supplement: Supplementary file 1 [file Presentation1.ZIP › Supplementary Figures.pdf]
