# Supplementary material for: Young Adults’ Sleep Duration on Work Days: Differences between East and West
Source: Front Neurol. 2014 May 28;5:81. doi: 10.3389/fneur.2014.00081 (PMC4036075; doi:10.3389/fneur.2014.00081)
Supplement: Supplementary file 1 [file Presentation1.ZIP › Supplementary Tables.pdf]

TABLE S1 Distribution of ethnicity in the UK sample

**Ethnicity**	**n**	**%**
European	430	69.0
African	66	10.6
Asian	56	9.0
Other	71	11.5

From only 623 participants in the UK sample were ethnicity data collected.

TABLE S2 Monthly distribution of participants in Singapore and the UK

	Singapore		UK	
**Month**	**n**	**%**	**n**	**%**
January	118	6.2	41	4.9
February	211	11.1	108	12.9
March	213	11.2	39	4.7
April	71	3.7	48	5.7
May	57	3.0	58	6.9
June	30	1.6	70	8.4
July	175	9.2	60	7.2
August	208	11.0	80	9.6
September	231	12.2	27	3.2
October	60	3.2	144	17.2
November	382	20.1	108	12.9
December	142	7.5	54	6.5

Percentages may not add up to 100% due to rounding.

TABLE S3 Differences in the natural light dark cycle between Singapore and the UK during standard and daylight saving times

	Singapore	UK		
	**Mean ± SE**	**Mean ± SE**	***t***	***p***
Duration of scotopic period (h)				
Standard time	11.93 ± 0.02	14.72 ± 0.11	25.49	< .001
Daylight saving time	11.85 ± 0.00	9.69 ± 0.01	16.55	< .001
Dusk time (local clock time)				
Standard time	19:09 ± 00:01	16:42 ± 00:04	36.42	< .001
Daylight saving time	19:08 ± 00:00	20:08 ± 00:04	13.73	< .001
Dawn time (local clock time)				
Standard time	07:05 ± 00:01	07:24 ± 00:03	6.21	< .001
Daylight saving time	06:59 ± 00:00	05:50 ± 00:04	18.99	< .001
Mid-scotopic time (local clock time)				
Standard time	01:07 ± 00:01	00:04 ± 00:01	43.19	< .001
Daylight saving time	01:03 ± 00:01	00:59 ± 00:01	6.84	< .001

Singapore does not have daylight saving time. The daylight saving time refers only to the UK.

SE = standard error

t = *t* value of independent-samples *t* tests

TABLE S4 Pearson correlations between natural light-dark cycle and sleep on work and free days in Singapore and the UK

**Natural light-dark cycle and sleep variables**	**Singapore**		**UK**	
	r	***p***	***r***	***p***
Duration of scotopic period and sleep				
Work days	-.03	.26	.00	.90
Free days	.01	.79	-.01	.74
Dusk time and bedtime				
Work days	**-.13**	< **.001**	-.03	.47
Free days	-.04	.10	-.05	.15
Dawn time and wake time				
Work days	-.04	.09	.02	.51
Free days	-.01	.82	.03	.45
Mid-scotopic time and mid-sleep time				
Work days	**-.08**	< **.001**	-.03	.32
Free days	-.01	.57	-.05	.14

Significant correlations are highlighted in bold.

TABLE S5 Contribution of country, type of day, morningness-eveningness preference, and the natural light dark cycle to sleep duration and timing for participants aged between 18 and 25 and in full-time education

	F	***p***	f^2
**Sleep duration**			
Country	**5.77**	**.02**	**.00**
Type of day	**59.71**	**< .001**	**.03**
Morningness-eveningness (ME) preference	1.27	.26	.00
Duration of scotopic period	.35	.55	.00
Country × Type of day	**5.43**	**.02**	**.00**
Country × ME preference	.72	.40	.00
Type of day × ME preference	**26.29**	**< .001**	**.01**
Country × Type of day × ME preference	.01	.92	.00
Gender	**5.47**	**.02**	**.00**
**Bedtime**			
Country	**5.24**	**.02**	**.00**
Type of day	**43.19**	**< .001**	**.02**
ME preference	**123.39**	**< .001**	**.07**
Dusk time	1.53	.22	.00
Country × Type of day	**3.97**	**.05**	**.00**
Country × ME preference	.57	.452	.00
Type of day × ME preference	**9.90**	**< .001**	**.01**
Country × Type of day × ME preference	.68	.41	.00
Gender	.63	.43	.00
**Wake time**			
Country	.03	.87	.00
Type of day	**157.77**	**< .001**	**.09**
ME preference	**149.40**	**< .001**	**.08**
Dawn time	.34	.56	.00
Country × Type of day	**6.87**	**.01**	**.00**
Country × ME preference	.00	.99	.00
Type of day × ME preference	**54.16**	**< .001**	**.03**
Country × Type of day × ME preference	.33	.57	.00
Gender	1.79	.18	.00
**Mid-sleep time**			
Country	**10.24**	**< .001**	**.01**
Type of day	**132.62**	**< .001**	**.07**
ME preference	**182.21**	**< .001**	**.10**
Mid-scotopic time	2.74	.10	.00
Country × Type of day	.42	.52	.00
Country × ME preference	.06	.81	.00
Type of day × ME preference	**40.73**	**< .001**	**.02**
Country × Type of day × ME preference	.63	.43	.00
Gender	.11	.74	.00

Significant effects are highlighted in bold. F and p values were derived from general linear mixed model analyses (refer to Methods for details). Effect sizes were indicated by Cohen's f^2. The reduced Morningness-Eveningness Questionnaire (rMEQ) score used was based on the 5-item version. Singapore: n = 1737; UK: n = 100.
